# Renal Hypoxia in CKD; Pathophysiology and Detecting Methods

**DOI:** 10.3389/fphys.2017.00099

**Published:** 2017-02-21

**Authors:** Yosuke Hirakawa, Tetsuhiro Tanaka, Masaomi Nangaku

**Affiliations:** Division of Nephrology, The University of Tokyo School of MedicineHongo, Japan

**Keywords:** chronic kidney disease, hypoxia, hypoxia-inducible factor, Nrf2, microelectrode, pimonidazole, BOLD-MRI, phosphorescence

## Abstract

Chronic kidney disease (CKD) is a major public health problem. Accumulating evidence suggests that CKD aggravates renal hypoxia, and in turn, renal hypoxia accelerates CKD progression. To eliminate this vicious cycle, hypoxia-related therapies, such as hypoxia-inducible factor (HIF) activation (prolyl hydroxylase domain inhibition) or NF-E2-related factor 2 activation, are currently under investigation. Clinical studies have revealed heterogeneity in renal oxygenation; therefore, the detection of patients with more hypoxic kidneys can be used to identify likely responders to hypoxia-oriented therapies. In this review, we provide a detailed description of current hypoxia detection methods. HIF degradation correlates with the intracellular oxygen concentration; thus, methods that can detect intracellular oxygen tension changes are desirable. The use of a microelectrode is a classical technique that is superior in quantitative performance; however, its high invasiveness and the fact that it reflects the extracellular oxygen tension are disadvantages. Pimonidazole protein adduct immunohistochemistry and HIF activation detection reflect intracellular oxygen tension, but these techniques yield qualitative data. Blood oxygen level-dependent magnetic resonance imaging has the advantage of low invasiveness, high quantitative performance, and application in clinical use, but its biggest disadvantage is that it measures only deoxyhemoglobin concentrations. Phosphorescence lifetime measurement is a relatively novel *in vivo* oxygen sensing technique that has the advantage of being quantitative; however, it has several disadvantages, such as toxicity of the phosphorescent dye and the inability to assess deeper tissues. Understanding the advantages and disadvantages of these hypoxia detection methods will help researchers precisely assess renal hypoxia and develop new therapeutics against renal hypoxia-associated CKD.

## Introduction

Chronic kidney disease (CKD) remains a major public health problem in both developed and developing countries despite enormous treatment efforts (Collins et al., 2015; Liyanage et al., 2015). Many clinical conditions, such as diabetes mellitus, glomerulonephritis, hypertension, and genetic disorders, are causative factors of CKD. However, once renal fibrosis, a major pathological hallmark of CKD, reaches a certain threshold, CKD progression becomes irreversible and independent of the initial cause (Fine et al., 2000; Nangaku, 2006). Thus, the final common pathways of CKD progression have been extensively studied. Renal hypoxia (i.e., decreased oxygen tension in the kidney), particularly in the tubulointerstitium, is a candidate prognostic marker for CKD progression (Nangaku, 2006; Mimura and Nangaku, 2010). Increased hemoglobin concentrations, which ameliorate renal hypoxia, are associated with a better renal prognosis in CKD patients (Tsubakihara et al., 2012). In support of hypoxia as a marker of poor renal function, sleep apnea syndrome and chronic obstructive pulmonary disease have been identified as risk factors for CKD (Iseki et al., 2008; Hanly and Ahmed, 2014; Chen and Liao, 2016). Although these studies are observational and no intervention studies with domiciliary oxygen therapy or continuous positive air pressure ventilation have been conducted, our results raise the possibility that conditions, such as SAS and COPD result in intermittent or continuous hypoxemia and increase the risk of CKD development via renal hypoxia aggravation.

## Cellular response to hypoxia

**Figure 1:**
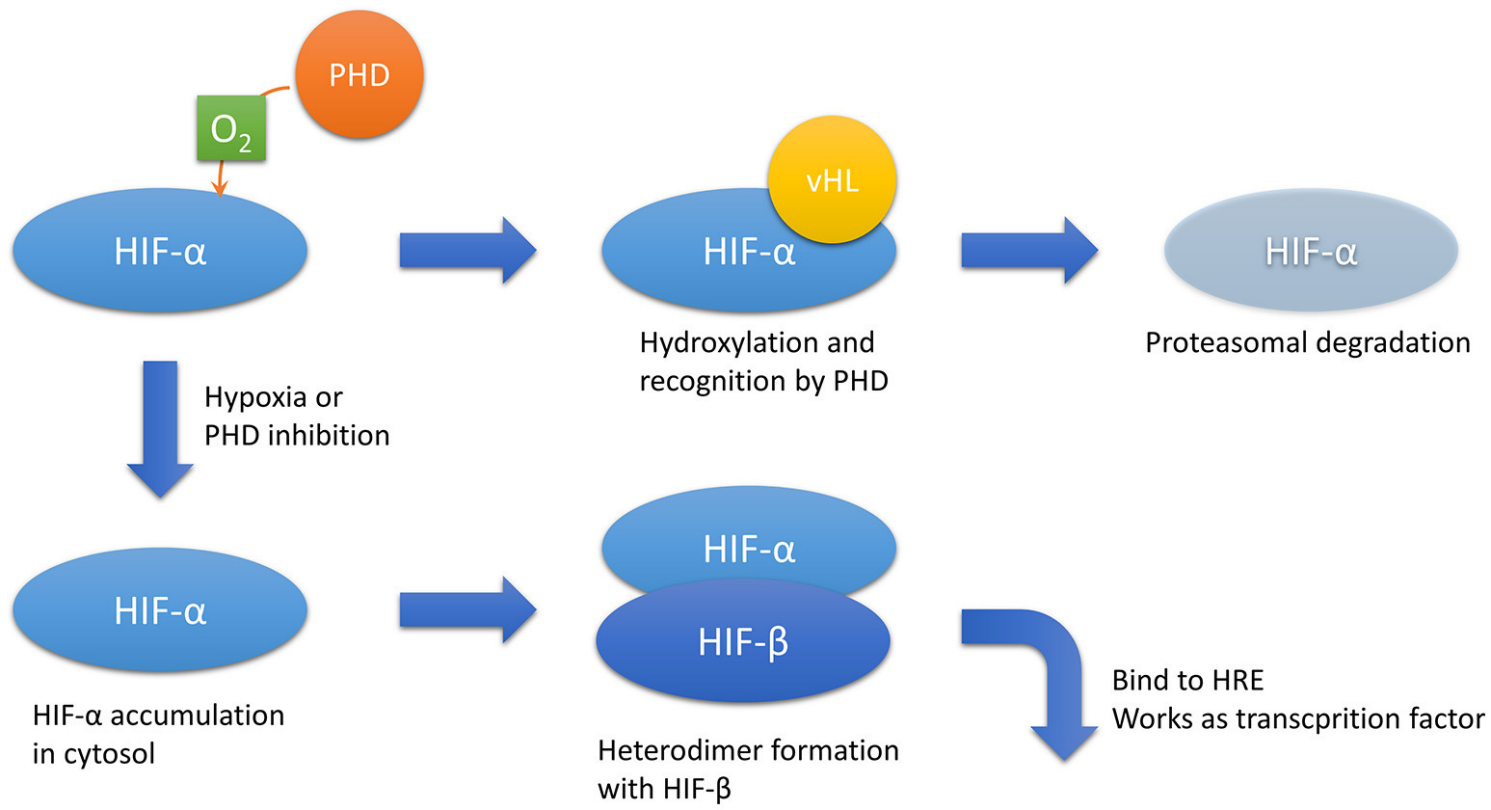
**HIF-α regulation**. In the presence of sufficient oxygen, HIF-α is hydroxylated by PHD. Hydroxylated HIF-α is recognized by vHL, which results in proteasomal degradation. In hypoxic conditions or PHD inhibition, HIF-α accumulates in the cytosol and forms a heterodimer with HIF-β, the hypoxia-insensitive unit. The heterodimer translocates to the nucleus and acts as a transcriptional factor that binds to HREs. Abbreviations: HIF, hypoxia-inducible factor; HREs, hypoxia-responsive elements; PHD, prolyl hydroxylase domain; vHL, von Hippel–Lindau disease tumor suppressor.

Oxygen is imperative for mitochondrial oxidative phosphorylation, a process that produces sufficient ATP amounts in metazoans. Thus, cellular response against insufficient oxygen supply is precisely controlled. Transcriptional factors known as hypoxia-inducible factors (HIFs) play a central role in the anti-hypoxia defense system (Semenza, 2014). HIFs are composed of two subunits: HIF-α, an oxygen-sensitive subunit, and HIF-β, an oxygen-insensitive subunit. The cellular concentration of HIF-α is strictly tuned by the oxygen tension. Prolyl hydroxylase domain (PHD) proteins hydroxylate HIF-α using oxygen molecules as substrates. Next, hydroxylated HIF-α recruits von Hippel–Lindau tumor suppressor, which triggers ubiquitination and proteasomal degradation (Figure 1). In this mechanism, HIF-α hydroxylation is the rate-limiting step. Therefore, the cellular concentration of HIF-α is dependent on oxygen tension. Given that HIF-α hydroxylation occurs in the cytosol, the intracellular oxygen tension is more important than the extracellular oxygen tension, which is usually estimated by measuring the intravascular oxygen tension.

Increases in cytoplasmic HIF-α concentrations due to decreased intracellular oxygen tension or PHD pharmacological inhibition lead to the formation of a heterodimer between HIF-α and HIF-1β. The heterodimer translocates into the nucleus and binds to the consensus enhancer via hypoxia-responsive elements (HREs), which then upregulates downstream target genes. The representative downstream genes include vascular endothelial growth factor (VEGF), erythropoietin (EPO), glucose transporter 1, and heme oxygenase 1, which protect the cell from hypoxia by promoting neovascularization, erythropoiesis, and glycolysis and by decreasing oxidative stress, respectively. There are three isoforms of HIF-α: HIF-1α, HIF-2α, and HIF-3α. In the kidney, HIF-1α is dominantly expressed in tubular cells, whereas HIF-2α accumulates in interstitial and endothelial cells (Rosenberger et al., 2002). The expression and function of HIF-3α remains elusive. Full-length HIF-3α works as a transcriptional factor with HIF-1β; however, some of its splice variants work as dominant negative forms against HIF-1α and HIF-2α (Duan, 2016).

## Renal hypoxia pathophysiology

The oxygen tension in the kidney is relatively lower in healthy individuals, even though the kidneys receive approximately 20% of the blood pumped out from the heart. One explanation is the oxygen shunt between the arterial and venous vessels that run parallel in the kidney, as proven by oxygen electrodes (Schurek et al., 1990). Recently, it has been actively debated whether an arteriovenous oxygen shunt affects renal oxygenation based on results from a three-dimensional computational model. To obtain a clear answer, the quantification of the geometry of artery–vein pairs in the kidney may be needed as results from the three-dimensional computational model vary with the assumption of background vascular networks (Ngo et al., 2014; Evans et al., 2015; Olgac and Kurtcuoglu, 2015). An oxygen shunt supposedly affects renal oxygenation and is likely accountable for the higher oxygen tension in renal veins than in efferent arterioles (Welch et al., 2001) and for the oxygen gradient in the kidney, i.e., the oxygen tension decreases deeper in the renal surface. The oxygen tension in the renal surface is approximately 50 mmHg, whereas the tension in the renal medulla is approximately 30 mmHg, as detected by microelectrodes (Friederich-Persson et al., 2013; Ow et al., 2014; Nordquist et al., 2015). A lower oxygen tension in normal kidneys has also been shown in a study that visualized HIF activity in transgenic mice (Safran et al., 2006). In these mice, the luciferase enzyme was fused to the oxygen-dependent degradation domain of HIF-1α. Thus, luciferase activity simulated HIF-α expression. Under normoxic conditions, the kidneys were the only organ that expressed sufficient luminescence that could be observed in the *in vivo* system.

In addition to its innate low oxygen tension, the kidneys suffer from severe hypoxia as CKD progresses. Renal hypoxia pathophysiology in CKD includes a decrease in oxygen supply and an increase in oxygen consumption. Loss of peritubular capillaries (PTCs) (Kang et al., 2001b; Ohashi et al., 2002), reduction in PTC flow (Matsumoto et al., 2004), and renal anemia (Astor et al., 2002) account for insufficient oxygen supply. Increases in oxygen demand in renal tubules have been shown in a reduced renal mass model (Harris et al., 1988; Nath and Paller, 1990), hypertensive model (Adler and Huang, 2002), and diabetes mellitus model (Korner et al., 1994), and this increase in the reduced renal mass model is corrected by renin–angiotensin–aldosterone axis blockade or HIF activation (Deng et al., 2009, 2010). These hypoxic changes likely exacerbate renal tubular damage. Outer medullary areas, including the S3 segment of the proximal tubules, medullary thick ascending limbs, and medullary collecting ducts, are considered to be most susceptible to hypoxia because histological changes are obvious in these segments of human kidneys with ischemia (Heyman et al., 2010). Differences in several features, including mitochondrial density and transport activity, account for the susceptibility to hypoxic insults. The medullary thick ascending limbs and medullary collecting ducts become more susceptible to hypoxic insults with forced tubular transport (Shanley et al., 1986; Brezis and Rosen, 1995; Epstein, 1997).

## Hypoxia-oriented therapy against CKD

Renal hypoxia accelerates CKD progression, and in turn, CKD exacerbates renal hypoxia. Therefore, eliminating this cycle is a promising strategy against CKD. One approach is HIF activation via PHD inhibition. Cobalt chloride is a classical PHD inhibitor, and it ameliorates CKD progression in a model of STZ-induced type 1 diabetes and Thy-1 nephritis and a remnant kidney model (Tanaka et al., 2005a,b; Nordquist et al., 2015). However, this treatment cannot be used in CKD patients because of severe adverse effects. PHD inhibitors that specifically inhibit HIF-α hydroxylation have recently been synthesized. These compounds are currently in clinical trials for renal anemia because the EPO gene is markedly upregulated by HIF-α stabilization (Fraisl et al., 2009; Maxwell and Eckardt, 2016), and several drugs show positive outcomes in clinical trials in non-dialysis CKD patients and dialysis patients (Besarab et al., 2016; Brigandi et al., 2016; Holdstock et al., 2016; Pergola et al., 2016; Provenzano et al., 2016). These drugs are expected to have protective effects against CKD.

## Oxidative stress-oriented therapy against CKD

**Figure 2:**
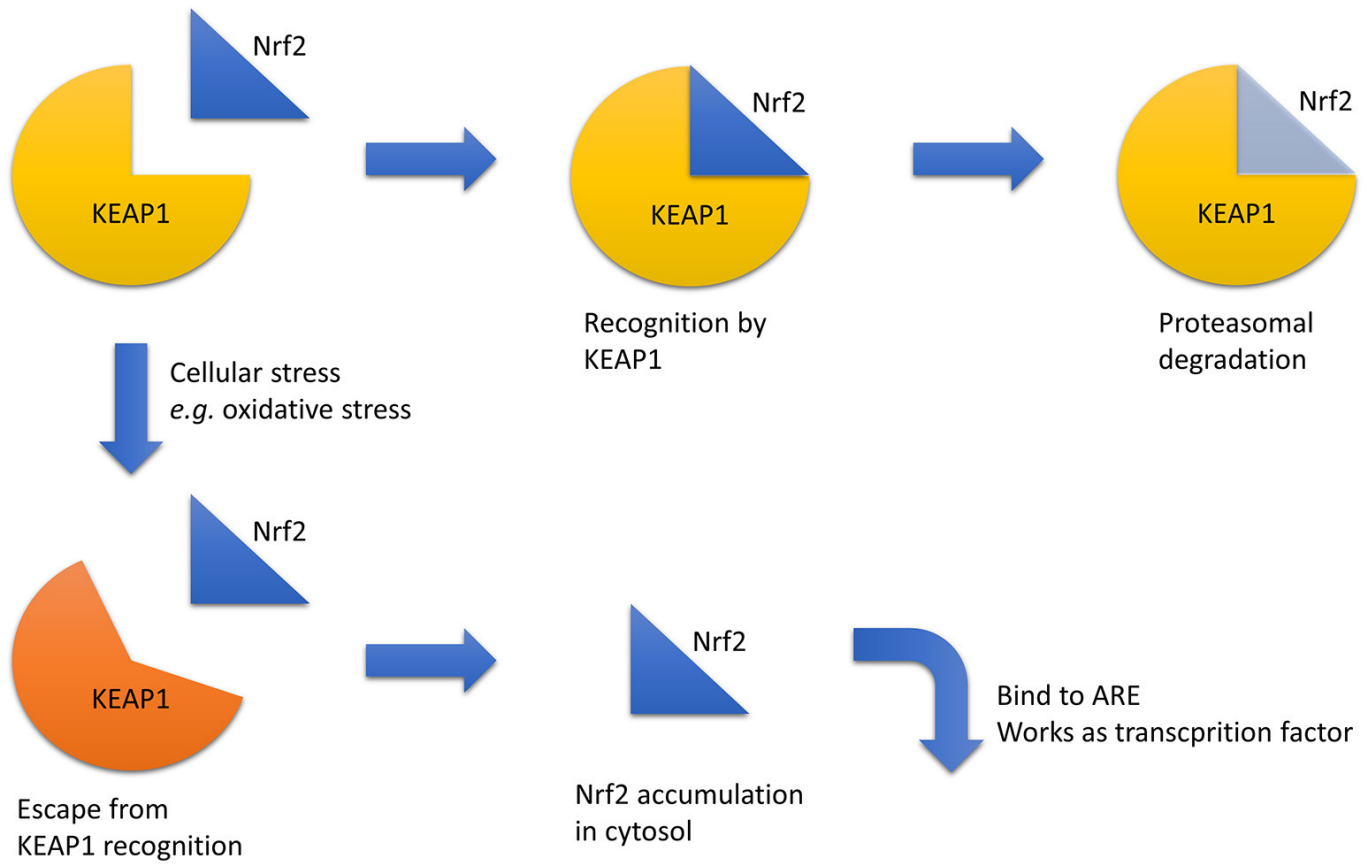
**Nrf2 regulation**. Under normal conditions, Nrf2 is recognized by KEAP1, which triggers its proteasomal degradation. In contrast, under stressful conditions, such as increased oxidative stress, Nrf2 recognition by KEAP1 is disturbed, and Nrf2 accumulates in the cytosol and acts as a transcriptional factor via ARE binding. Abbreviations: ARE, anti-oxidant responsive element; KEAP1, Kelch-like ECH-associated protein 1; Nrf2, NF-E2 related factor 2.

Another treatment approach is the enhancement of the anti-oxidant response because hypoxia is closely related to increased oxidative stress. One relevant transcriptional factor for this approach is NF-E2 related factor 2 (Nrf2). Nrf2 is degraded via recognition by Kelch-like ECH-associated protein 1 (Keap1) in the cytosol under normal conditions. When certain chemical stresses, such as oxidative stress, are induced, Nrf2 escapes degradation, translocates into the nucleus, binds to anti-oxidant responsive elements (AREs), and upregulates downstream genes (Bryan et al., 2013; Figure 2). Nrf2–ARE axis activation induces strong anti-oxidative effects; therefore, the pharmacological activation of the Nrf2–ARE pathway is a promising target for various diseases, including kidney diseases. Bardoxolone methyl, an Nrf2 activator via Keap1 inhibition, increases eGFR in CKD patients with diabetes mellitus; however, the change in eGFR is not considered primary or secondary outcomes, and hypertension and an increased urinary albumin-to-creatinine ratio was seen in the bardoxolone group (Pergola et al., 2011; de Zeeuw et al., 2013). However, a high frequency of cardiovascular events in the bardoxolone methyl group led to the termination of the phase III trial (de Zeeuw et al., 2013). A subsequent analysis revealed that most of these patients suffered from excessive fluid retention at the early stage. Considering that these clinical signs can be predicted under close follow-up of the patients and that diabetic kidney disease is major cause of end-stage renal disease, the potential benefit of this drug was reconsidered and a new phase II trial that excludes patients with a high risk of cardiovascular disease is ongoing in Japan.

These hypoxia-targeting pharmaceutical therapies are promising. Theoretically, these therapies are effective only on patients whose kidneys are truly hypoxic. The renal oxygen state widely varies in CKD patients (Michaely et al., 2012). Thus, it is important to identify patients whose kidneys are sufficiently hypoxic, i.e., more responsive to hypoxia-oriented therapies. However, the currently available hypoxia detection methods have serious disadvantages. Thus, there is a compelling need for a better understanding of hypoxia detection methods.

## General principles of oxygen tension assessment

The most important function of oxygen molecules is their action as an oxidant. In addition, oxygen can bind to hemoglobin to increase its solubility in blood. A lower oxygen tension results in HIF–HRE axis activation. Thus, we consider that methods to measure the oxygen tension can be divided into four groups: (i) assessment of the oxidative power of oxygen molecules (Clark, 1956; Arteel et al., 1995), (ii) measurement of the oxyhemoglobin/total hemoglobin ratio (Prasad et al., 1996), (iii) detection of HIF activity (Safran et al., 2006), and (iv) direct measurement of oxygen molecules by the assessment of fluorescence or phosphorescence quenching rate (Rumsey et al., 1988; Mik et al., 2006). Although these four methods unequivocally depend on the oxygen tension, there are some confounding factors in their interpretation. For example, when the oxidative power of oxygen molecules is measured, other oxidants or reductants can influence the result. Notably, oxidative stress levels increase in CKD patients (Himmelfarb et al., 2002). Although the oxyhemoglobin/total hemoglobin ratio is clinically used to determine a patient's general oxygenation state, this value can be affected by the partial pressures of oxygen and carbon dioxide, changes in pH, and other micro-environmental changes (Bohr effect). Blood oxygen level-dependent magnetic resonance imaging (BOLD-MRI) employs hemoglobin as an oxygen sensor; however, this method only measures the deoxyhemoglobin concentration and not the deoxyhemoglobin/total hemoglobin ratio. Thus, this method is influenced by the hemoglobin concentration, which cannot be precisely measured in small veins (Prasad et al., 1996). Renal anemia and acid–base disorders frequently occur in CKD patients, which may explain why the use of renal BOLD-MRI in CKD patients remains controversial (Inoue et al., 2011; Michaely et al., 2012; Pruijm et al., 2014). HIF activation may be influenced by factors other than oxygen molecules, such as erythropoietin and indoxyl sulfate (Chiang et al., 2011). Indoxyl sulfate, a representative uremic toxin, suppresses *EPO* gene transcription in hypoxic HepG2 cells in an HIF-dependent manner, and the oral administration of indole, a precursor of indoxyl sulfate, decreases the serum erythropoietin concentration in rats.

Another important consideration is whether the method employed reflects the intracellular or extracellular oxygen tension. As previously noted, the HIF-mediated cellular response against hypoxia is regulated by the hydroxylation of HIF-α, which uses oxygen as a substrate. Thus, the intracellular oxygen tension is important. For example, anemia can cause dissociation between the intracellular and extracellular oxygen tensions because anemia does not change the partial pressure of oxygen, but rather changes the total oxygen amount in arterial blood (i.e., decrease in hemoglobin concentration). This condition should not be overlooked because renal anemia development is associated with renal hypoxia in CKD patients.

Another important aspect to consider is the generation of quantitative or qualitative data. Microelectrodes, BOLD-MRI, pimonidazole protein adduct immunohistochemistry, and HIF activation detection are frequently used as hypoxia detection methods (Heyman et al., 2008). Phosphorescence lifetime measurement is a relatively novel intravital oxygen sensing technique; thus, we have included this technique in our review (Parpaleix et al., 2013; Spencer et al., 2014; Hirakawa et al., 2015). The characteristics of these methods are summarized in Table 1. For clinical applications, BOLD-MRI is the only appropriate method. For animal experiments, microelectrodes are a gold standard for oxygen tension measurement, although the specific measurement site is unclear. Pimonidazole staining and HIF activation detection can be used for paraffin sections and has the advantage of spatial resolution. Phosphorescence lifetime measurement is a highly sensitive method, but it requires special instrumentation.

**Table 1:** **Comparison of hypoxia detection methods**.

**Method**	**What is measured?**	**Intracellular/extracellular**	**Qualitative/quantitative**	**Clinical applicability**
Pimonidazole staining	Oxidative power of O_2_	Intracellular	Qualitative	PET probe only
Microelectrode	Oxidative power of O_2_	Extracellular	Quantitative	No
Detection of the activated HIF–HRE axis	Activation of the HIF–HRE axis	Intracellular	Qualitative	No
BOLD-MRI	Deoxyhemoglobin concentration	Extracellular	Quantitative	Yes
Phosphorescence lifetime measurement	Quenching by an oxygen molecule	Depends on dyes	Quantitative	No

*BOLD-MRI, blood oxygen level-dependent magnetic resonance imaging; HIF, hypoxia-inducible factor; HRE, hypoxia-responsive element*.

Comparison of results obtained via different methods is attractive, and there is a report that compared pO_2_ measured by a microelectrode with that measured by a fluorescence needle probe in the kidney and which mentions that there is a slight difference in outcomes between these two methods (O'Connor et al., 2006). However, this comparison cannot determine which of the two methods is reliable; the calibration method of each method differs, and the measurement site is not always the same. Therefore, the comparison of pO_2_ obtained via different techniques needs to be interpreted with that consideration in mind.

## Hypoxia detection methods

### Microelectrodes

The use of microelectrodes is a classical technique and the gold standard for determining the oxygen tension in living cells and animals. The principle of this technique is based on oxidation–reduction reactions. The reaction for a silver electrode is shown below:

(1) $$4\text{Ag}+4{\text{Cl}}^{-}\;\to 4\text{AgCl}+4{\text{e}}^{-}\cdot \cdot \cdot$$

(2) $$4{\text{H}}^{+}+4{\text{e}}^{-}+{\text{O}}_{2}\;\to 2{\text{H}}_{2}\text{O}\cdot \cdot \cdot$$

This reaction contains an oxygen molecule as a reactant in Equation 2; thus, the electric current is dependent on the oxygen concentration, assuming that the voltage is constant. This method is widely used to determine renal oxygenation as well as other visceral organ oxygenation, such as the liver. The kidney has a natural oxygen gradient; therefore, data obtained from a microelectrode is dependent on depth. Typically, representative oxygen tensions in the renal cortex and medulla are 50 and 30 mmHg, respectively. This ability to distinguish between the renal cortex and the medulla is an advantage of the microelectrode; however, technical proficiency is required to accurately measure medullary oxygen tension. This technique has two major disadvantages, in addition to its non-applicability in humans. First, this method is highly invasive. The technique requires renal capsule removal and needle insertion into the renal tissue, which results in microbleeding. This method intrinsically hinders repeat measurements within an individual. The second disadvantage is that microelectrodes measure the partial pressure of oxygen from both microcirculation and microbleeding based on the fact that a microelectrode is slightly pulled after insertion to prevent compression. Despite these disadvantages, the use of microelectrodes remains the gold standard for determining tissue oxygen tension because of its superior quantitative performance.

To overcome the disadvantages of this established method, novel modifications have been developed. The first involves the use of telemetry (Koeners et al., 2013; Emans et al., 2016). For oxygen tension telemetry, carbon paste electrodes are used to avoid surface poisoning (Bolger et al., 2011). Once the electrodes are implanted, renal oxygenation can be monitored for 2 weeks without anesthesia. This technique resolves the two major disadvantages inherent to the conventional microelectrode method, including the continuous measurement within an individual and the avoidance of acute effects from general anesthesia. Another approach is urinary oxygen tension measurement (Evans et al., 2014). Urinary oxygen tension reflects renal medullary oxygen tension and is useful for monitoring renal oxygenation in critically ill patients (Kainuma et al., 1996; Morelli et al., 2003). This technique does not require intervention other than the insertion of electrode-equipped bladder catheters; thus, it has the advantage of being easily applied in clinical settings. One disadvantage is the general lack of proof of this in smaller animals. However, a recent study in rabbits reported the ability of urinary oxygen tension measurements to reflect renal medullary oxygen tension (Sgouralis et al., 2016).

### Nitroimidazole probes

Pimonidazole is a nitroimidazole. Nitroimidazoles are hypoxic markers that were initially used to detect hypoxic tumors, which are frequently resistant to radiotherapy. Pimonidazole was used to detect tissue hypoxia in a physiological range (Arteel et al., 1995). Under normoxic conditions, pimonidazole is oxidized by oxygen molecule, whereas pimonidazole is reductively activated under hypoxic conditions. Reduced pimonidazole binds to intracellular thiol-containing proteins. Pimonidazole protein adducts are only detected in hypoxic cells after systemic administration. The detection of pimonidazole protein adducts is typically achieved by immunohistochemistry. Thus, this technique requires the systemic administration of pimonidazole and the preparation of paraffin-embedded sections or frozen sections, which prohibits its use in living animals. Another disadvantage is that this technique reflects the redox state; thus, a redox imbalance independent of the oxygen tension can influence the result. In the kidneys, pimonidazole-positive areas are naturally observed in the medulla and corticomedullar junction (Manotham et al., 2004; Fong et al., 2016), which supports the idea that these areas are more hypoxic than the superficial cortex. In CKD models, the pimonidazole-positive area expands (Manotham et al., 2004; Tanaka et al., 2006). This technique can only detect areas with oxygen tensions lower than a certain threshold (approximately below 10 mmHg); thus, it is neither quantitative nor indicative of the oxygen tension.

To overcome the disadvantage of paraffin-embedded sections, several positron emission tomography (PET) tracers with nitroimidazoles have been studied. For example, ^18^F-fluoroazomycin arabinoside (^18^F-FAZA) (Piert et al., 2005) was found to accumulate in hypoxic tumors. This probe can be administered in clinical settings, and several papers have proved that it is useful for prognostic prediction in non-small-cell lung cancer (Saga et al., 2015; Kinoshita et al., 2016). It remains unclear if this method can successfully detect renal hypoxia in healthy individuals or CKD patients. The difficulty in detecting renal hypoxia is based on the fact that the oxygen tension of non-tumorous organs, including the kidney, is likely to be much milder than that of tumor tissues, even in pathological conditions (Handley et al., 2011). Pimonidazole binds to intracellular thiols at oxygen tensions below 10 mmHg, which is frequently lower than the tensions found in non-tumorous tissues. Another disadvantage is that ^18^F-FAZA is physiologically distributed to even normal kidneys and is excreted from the kidney like other PET tracers (Koh et al., 1992). In PET imaging, there is limited resolution with which to distinguish PET probes in the urinary space and intracellular PET probes. However, PET tracing using nitroimidazoles is potentially preferred because this technique is less invasive and suitable for use in CKD patients. Furthermore, this probe reflects the intracellular rather than the extracellular oxygen tension. The optimization of the oxygen tension threshold and biokinetics of PET tracers may overcome the disadvantages listed above and result in new information on renal hypoxia in CKD patients.

### HIF activation detection

The cellular response against hypoxia is mainly regulated by HIF; therefore, HIF activation can be used as evidence on tissue hypoxia. One detection method assesses HRE-downstream genes or proteins in tissues. The advantage of this method is that several HIF target proteins are hypoxia markers and affect CKD progression. An example of one of these targets is VEGF. The transcription of the *VEGF* gene is strongly enhanced under hypoxic conditions. Exogenous VEGF administration ameliorates renal fibrosis in a remnant kidney model, whereas sunitinib administration, a VEGF receptor inhibitor, worsens renal injury in this model (Kang et al., 2001a; Machado et al., 2012), although sunitinib also blocks the platelet-derived growth factor receptor. The expression of HIF target genes can be affected by other transcriptional factors and thus is not always affected by the oxygen tension alone. The protein level of VEGF can remain unchanged in some CKD models despite renal hypoxia (Futatsugi et al., 2016).

Classical techniques, such as reverse transcription PCR and immunohistochemistry, require tissue sample preparation. Another method is the *in vivo* imaging of HIF activity using luciferase. Our group established hypoxia-sensing transgenic rats, in which the luciferase gene was controlled by tandem repeats of HREs located in the 5′ UTR of the *VEGF* gene (Tanaka et al., 2004). A similar approach was employed by Safran et al. who established a hypoxia-sensing mouse strain that expressed firefly luciferase fused to the HIF-1α protein (Safran et al., 2006). These animals emit luminescence after the administration of a systemic chemiluminescent substrate, and the luminescence can be measured from outside the body. This strain was used to detect a decrease in HIF-α hydroxylation induced by hypoxemia and pharmacological PHD inhibition. The advantage of this technique is the lack of autoluminescence in the body, whereas a disadvantage is the reabsorption of luminescence. The kidney contains a significant amount of blood; thus, it can absorb light at an optical wavelength. It is unclear if luminescence from the kidney truly reflects total renal luciferase activity or only superficial activity. Kuchimaru et al. may have overcome this disadvantage (Kuchimaru et al., 2016). They succeeded in synthesizing a novel luminescent substrate whose wavelength was considerably longer (λ_*max*_ = 677 nm) than classical luciferins. Owing to its near-infrared wavelength, this substrate can be used to observe deeper tissue and will definitely aid in the intravital imaging of HIF activity.

### MRI-based assessment

BOLD-MRI is a useful tool to evaluate renal oxygenation. Here, the spin–spin relaxation rate R2* is measured, which reflects the deoxyhemoglobin concentration in veins (Prasad et al., 1996). To use this technique for tissue oxygenation approximation, we must speculate three proportional relationships: the deoxyhemoglobin concentration and deoxyhemoglobin/total hemoglobin ratio, deoxyhemoglobin/total hemoglobin ratio and partial pressure of oxygen in veins, and partial pressure of oxygen in veins and tissue oxygen level. These three proportional relationships are confounded by the hemoglobin concentration in veins, change in the oxygen affinity of hemoglobin, and intracellular/extracellular oxygen tension dissociation, respectively. Renal anemia and systemic acid–base disorders frequently occur in CKD patients, which may change the oxygen affinity of hemoglobin. The existence of an oxygen shunt in renal arterial and venous vessels suggests that the oxygenation of renal venous vessels is independent of tissue oxygenation. These disadvantages must be taken into account when interpreting data from BOLD-MRI of the kidney.

Despite these disadvantages, BOLD-MRI is a promising method because it is a non-invasive technique, particularly when applied in humans. A previous report has shown that the renal R2* value correlated with eGFR in CKD patients without diabetes (Inoue et al., 2011), whereas another report did not find this correlation in CKD patients (Michaely et al., 2012). The discrepancy between these two reports may be due to differences in patient backgrounds. Another report has indicated there are several factors other than the oxygen tension, such as blood glucose and uric acid levels, that influence the result of BOLD-MRI, whether directly or indirectly (Pruijm et al., 2014). It is debatable whether BOLD-MRI can properly address the heterogeneity of CKD patients.

BOLD-MRI, an R2*-based technique, employs hemoglobin as an oxygen sensor. However, another MRI-based technique that directly measures oxygen molecules has been recently reported (O'Connor et al., 2009; Winter et al., 2011). In this technique, the relaxation rate of the T1 signal (R1) is measured, which is related to the oxygen molecule itself. One report has suggested that R1 in lipids changes with the oxygen tension in mice tumors, whereas another report has shown that the R1-based technique is less sensitive in detecting renal hypoxia in hypoxemia than the R2*-based technique (Jordan et al., 2013; Ganesh et al., 2016). Together with the low invasiveness of MRI-based techniques and the limitation of BOLD-MRI, R1-based measurement is a potentially viable method because it directly determines the oxygen tension.

### Phosphorescence

Phosphorescence is a type of light emitted from a molecule in the triplet excited state. Molecules in this state can lose their energy by being hit by oxygen molecules. Phosphorescence intensity and phosphorescence lifetime depend on the oxygen concentration (Rumsey et al., 1988). Thus, this technique can directly measure the oxygen tension. Substances that emit phosphorescence are limited, and exogenous phosphorescence dyes are used to measure phosphorescence. Notably, the phosphorescence lifetime is far longer than the fluorescence lifetime. Both phosphorescence intensity and phosphorescence lifetime can be quantitatively measured. However, phosphorescence dye concentrations in the target organ are required to determine the oxygen tension *in vivo* using the phosphorescence intensity. Therefore, phosphorescence lifetime measurements are frequently used to determine the oxygen tension *in vivo* (Mik et al., 2008; Parpaleix et al., 2013; Spencer et al., 2014; Hirakawa et al., 2015). The phosphorescence lifetime does not depend on dye concentration as long as it sufficiently accumulates. In phosphorescence lifetime measurements, the Stern–Volmer equation is used:

(3) $$\left[{\text{O}}_{2}\right]=\frac{1}{k_{q}}\left(\frac{1}{\tau }-\frac{1}{\tau_{0}}\right)\cdot \cdot \cdot$$

where k_*q*_ is the rate constant, τ is the phosphorescence lifetime at the oxygen concentration of [O_2_], and τ_0_ is the phosphorescence lifetime in an anoxic condition (Vanderkooi et al., 1987). These two constants, k_*q*_ and τ_0_, depend on the phosphorescence dye and circumstantial microcondition, such as solvent. Thus, the determination of these parameters is problematic.

The oxygen dependence of the phosphorescence lifetime was established in the 1980s. It is utilized with fluorescence needle probes, which contain an oxygen-sensitive fluorescence dye, such as protoporphyrin IX (PpIX) described below, which has similar characteristics against oxygen. Several studies have employed this fluorescence needle probe because of its high sensitivity to the oxygen tension (O'Connor et al., 2006; dos Santos et al., 2007). However, this method cannot overcome the two major disadvantages of microelectrodes: unclear measurement site and high invasiveness. Thus, another way to apply phosphorescence lifetime measurement to *in vivo* O_2_ measurement is warranted, and currently, the administration of phosphorescence dyes that are distributed in a certain tissue or cell is frequently used. One research milestone was the report by Spencer et al. (2014). They employed a water-soluble phosphorescence dye, PtP-C343, to measure the oxygen tension in arterioles penetrating cortical bones and showed that the oxygen tension decreased when the arteriole entered the medullary canal in mice. This research directly proved the lower oxygen tension in the bone marrow, which was hypothesized based on the unique vasculature system in the marrow and pimonidazole protein adduct immunohistochemistry (Suda et al., 2011; Nombela-Arrieta et al., 2013).

Powerful research on the kidney was published by Mik et al. (2008). They used Oxyphor G2 as a water-soluble phosphorescence dye and proved that oxygen tension in renal vein was decreased after endotoxin shock in rats. This work clarified where the oxygen tension should be measured (i.e., in renal veins). The phosphorescence dye is excited only in blood in renal vein. In this study, there is no phosphorescence signal from arterial blood, capillary blood, or interstitial fluid, which is problematic in the study of solid organs.

The aforementioned phosphorescence dyes are distributed in the extracellular fluid, mainly in the blood; thus, these dyes cannot directly determine the intracellular oxygen tension. Our group recently reported a novel technique for measuring the intracellular oxygen tension using BTPDM1, a cationic lipophilic phosphorescence dye (Yoshihara et al., 2015). In the kidney, this dye is found only inside tubular cells. Thus, phosphorescence lifetime imaging reflects only the intracellular oxygen tension in tubules. Using this method, phosphorescence lifetime measurement revealed renal hypoxia in CKD, and the phosphorescence lifetime was successfully converted to the partial pressure of oxygen using the calibration curve obtained from a cultured tubular cell line. One disadvantage of this technique is dye toxicity when applied in human beings because BTPDM1 contains iridium, a heavy metal. Another dye that may overcome this disadvantage is PpIX (Mik et al., 2006). PpIX is a precursor of heme and is unique in that it can emit delayed fluorescence. This delayed fluorescence is similar to phosphorescence in terms of oxygen sensitivity, i.e., its phosphorescence lifetime depends on the oxygen tension. PpIX localizes to the mitochondria, and its physiological concentration is so low that it cannot be used as an endogenous oxygen sensor. However, when a large amount of 5-aminolevulinic acid (5-ALA), a PpIX precursor, is administered, the cellular synthesis of PpIX sufficiently increases to allow its use as an oxygen sensor via delayed fluorescence lifetime measurements. Using this method, the topical application of 5-ALA enables PpIX accumulation in the skin of healthy human volunteers and the oxygen tension estimate in human skin (Harms et al., 2013). Thus, this technique can be applied to abdominal organs, such as the liver (Bodmer et al., 2012). These phosphorescence or delayed fluorescence lifetime measurement techniques have the advantage of direct oxygen measurements and a superior quantitative performance. In contrast, these techniques cannot be used for assessing the deeper portion of the kidney, such as the outer medullary region, because phosphorescence is absorbed in tissues, particularly by hemoglobin.

## Conclusion and future perspectives

In this review, we addressed the pathophysiological importance of renal hypoxia with a focus on hypoxia detection. Although oxygen tension determination has been studied for a long time, recent advances have made it possible to achieve continuous measurements, non-invasive oxygen assessments, and intracellular oxygen assessments. All these methods have their advantages and disadvantages; therefore, a comprehensive understanding and proper selection of the available methods are required to assess renal hypoxia and identify the final common pathway in individual hypoxia patients. Thus, a sufficient understanding of oxygen detection techniques will help researchers develop new drugs against CKD and renal hypoxia.

## Author contributions

YH wrote the manuscript and TT and MN edited the manuscript.

### Conflict of interest statement

The authors declare that the research was conducted in the absence of any commercial or financial relationships that could be construed as a potential conflict of interest.
